# Which EDE-Q items are structurally relevant in borderline personality disorder? An exploratory graph analysis

**DOI:** 10.1186/s40479-026-00355-1

**Published:** 2026-06-29

**Authors:** Nora M. Laskowski, Hannah R. Hambruch, Isa Häkkinen, Iris Pollmann, Claudia M. Deutschmann, Gerrit Brandt, Cristina Ballero Reque, Georgios Paslakis

**Affiliations:** 1 Department of Psychosomatic Medicine and Psychotherapy, LWL- University Hospital, Ruhr-University Bochum, Bochum, Germany; 2 Clinic for Psychosomatics and Psychotherapy, Center for Integrative Psychiatry ZIP gGmbH, Kiel Campus, Kiel, Germany

**Keywords:** Borderline personality disorder, Eating disorders, EDE-Q, Exploratory graph analysis, Factor structure, Body dissatisfaction, Psychometrics, EGA

## Abstract

**Background:**

Borderline personality disorder (BPD) frequently co-occurs with eating disorders (EDs). Despite this clinical relevance, the dimensional structure of commonly used assessment instruments, such as the Eating Disorder Examination Questionnaire (EDE-Q), has not been sufficiently examined in BPD populations. Previous research suggests that the original four-factor structure of the EDE-Q may not be stable across different populations.

**Methods:**

This study examined the structure of the EDE-Q in a clinical sample of individuals with BPD (*N* = 73) using Exploratory Graph Analysis (EGA), a network-based approach to dimensionality assessment. Dimensionality and item stability were evaluated using nonparametric bootstrapping. Robustness was assessed via sensitivity analyses, including median imputation, variation of the regularization parameter, comparison of community detection algorithms, and leave-one-item-out procedures.

**Results:**

After iterative item reduction, a three-dimensional solution emerged, comprising 13 items. The identified dimensions reflected (1) *Restraint*, (2) *Cognitive–Interpersonal Eating Concerns*, and (3) *Body Dissatisfaction*. The final solution demonstrated high robustness across all sensitivity analyses, with identical dimensional structures and item assignments under multiple conditions.

**Conclusions:**

The findings suggest that the original four-factor structure of the EDE-Q may not fully generalize to individuals with BPD. Several aspects of the identified structure are consistent with previous research reporting deviations from the original EDE-Q model. These findings underscore the importance of validating assessment instruments within specific clinical populations and support a cautious interpretation of standard EDE-Q subscale scores in individuals with BPD.

## Background

Borderline personality disorder (BPD) is a severe and complex psychiatric condition characterized by pervasive instability in affect regulation, self-image, and interpersonal relationships, as well as impulsivity and self-destructive behaviours [1]. Prevalence estimates vary across studies, with a global pooled rate of approximately 1.8% [2]. In clinical settings, prevalence is substantially higher, with reported rates of 22.4% among inpatients and 11.8% among outpatients [3].

According to the fifth edition of the Diagnostic and Statistical Manual of Mental Disorders (DSM-5), a diagnosis requires the presence of at least five out of nine symptoms, including frantic efforts to avoid real or imagined abandonment, unstable interpersonal relationships and self-image, impulsivity, self-harm, affective instability, chronic feelings of emptiness, inappropriate anger, and dissociation [1]. However, symptom presentation in BPD is highly heterogeneous and is associated with substantial variability in clinical risk and functional impairment [4, 5].

Co-occurring psychiatric conditions are highly prevalent in BPD and contribute to its clinical complexity and poor prognosis. Common comorbidities include mood, anxiety, substance use, and other personality disorders [6–9]. Eating disorders (EDs) are also particularly frequent, with approximately 29.7% of individuals with BPD meeting diagnostic criteria for an ED at some point in their lives [10]. Commonly reported subtypes include binge-eating disorder (BED; 16.3%), bulimia nervosa (BN; 16.3%), anorexia nervosa (AN; 9.98%) and DSM-IV ED not otherwise specified (EDNOS; 18.8%) [10], although this category reflects historical diagnostic nomenclature from studies using earlier classification systems. The co-occurrence of BPD and EDs is associated with greater symptom severity, increased impulsivity, depressive symptoms and suicidality, and has also been linked to poorer treatment outcomes and longer inpatient stays in ED populations [11–13].

Transdiagnostic processes such as emotional dysregulation and impulsivity have been implicated in both BPD and EDs and may contribute to their co-occurrence [14–16]. These processes appear to be particularly relevant in bulimic and purging-related ED subtypes, which show stronger associations with BPD than restrictive ED presentations [15]. In addition, the co-occurrence of BPD and EDs has been associated with an elevated risk of self-harm and suicidality [15, 16]. Disturbances in self-concept characteristic of BPD, including identity instability and difficulties in self-acceptance, may also contribute to eating- and body-related difficulties [17]. Research further suggests that fear of abandonment may represent and additional mechanism linking BPD and disordered eating behaviours with ED symptoms potentially serving as maladaptive strategies to manage affective or interpersonal distress [18].

Given these shared mechanisms and the clinical relevance of co-occurring EDs, accurate assessment of disordered eating in BPD is essential. However, relatively little is known about whether commonly used measures of ED pathology adequately capture eating-related symptom structures in individuals with BPD. Instruments such as the Eating Disorder Examination Questionnaire (EDE-Q [19]) have largely been developed and validated in general or ED populations, and their applicability within BPD populations remains unclear.

The EDE-Q [19] is one of the most widely used self-report instruments for assessing ED psychopathology in both research and clinical practice. It captures core cognitive and behavioural features across four subscales: *Restraint*, *Eating Concern*, *Shape Concern*, and *Weight Concern*. However, a growing body of research has questioned the stability and validity of this original four-factor structure across diverse populations, including men and gender minority individuals [20, 21]. Several alternative factor structures have been proposed [22–25], and a recent systematic review concluded that empirical support for the original model is limited, with evidence suggesting that revised or shortened versions may better capture core aspects of restraint and body-related concerns [26]. Notably, research applying Exploratory Graph Analysis (EGA) has demonstrated that the dimensional structure of the EDE-Q varies across clinical ED groups, further challenging the assumption of a stable underlying factor structure [27]. The dimensional structure of the EDE-Q has not yet been examined in individuals with BPD.

To address this gap, the present study applied EGA [28], a network-based approach to dimensionality assessment, to investigate the structure of the EDE-Q in a clinical sample of individuals with BPD. Compared to traditional latent variable approaches such as exploratory or confirmatory factor analysis, EGA has demonstrated comparable or superior accuracy in identifying dimensional structures and offers additional advantages, including bootstrap-based evaluation of dimensional and item stability [28–30]. Importantly, EGA may be particularly well suited for BPD populations, which are characterized by heterogeneous and dynamically interacting symptom profiles that may not conform to a strictly latent variable structure. Network approaches allow for the identification of symptom communities based on empirical associations, thereby accommodating transdiagnostic and potentially non-latent organization of psychopathology. Prior research further suggests that cross-sectional network structures capture meaningful information about symptom dynamics, as associations between symptoms have been shown to predict relationships between change trajectories in BPD [31]. Moreover, network approaches can reveal stable and interpretable symptom communities, providing insight into the organization and potential mechanisms underlying psychopathology [31, 32].

Although self-report measures are widely used in ED research, the dimensional structure of the EDE-Q has not yet been examined in individuals with BPD. Given the high co-occurrence of ED pathology in this population, it remains important to investigate how eating-related symptoms are organized within individuals with BPD. Therefore, the present study aimed to explore the dimensional structure of the EDE-Q in individuals with BPD using EGA, with a particular focus on the stability and interpretability of its underlying dimensions. Based on prior research demonstrating inconsistent support for the original structure across different populations, we tentatively expected deviations from the original four-factor structure.

## Methods

### Procedure and population

Between May 2021 and December 2024, a total of 80 adult patients with a confirmed diagnosis of BPD were recruited as part of a larger study [33]. Inclusion criteria further required legal adulthood. Individuals presenting with acute severe depression, suicidality, or psychosis were excluded. Participation was voluntary and anonymous, and written informed consent was obtained prior to data collection. Patients were recruited during Dialectical Behaviour Therapy (DBT), either as inpatients (*n* = 70) at the University Clinic for Psychosomatic Medicine and Psychotherapy, Ruhr-University Bochum (Luebbecke, Germany), or as outpatients/patients of the day hospital program (*n* = 10) at the Centre for Integrative Psychiatry (Kiel Campus, Germany). The present study represents a secondary analysis of data collected within the original study [33]. The study was conducted in accordance with ethical guidelines and was approved by the Ethics Committee of the Medical Faculty of the Ruhr-University Bochum (AZ 2021 − 743_2).

Participants completed a set of validated questionnaires (see below) at varying time points during DBT treatment, as no standardized assessment time point was specified within the original study. All participants provided sociodemographic information, including gender (male, female, diverse), education level (less than or more than 12 years of schooling), age, weight, height, and prior psychotherapy experience before DBT admission. Body mass index (BMI) was calculated based on weight and height. Information on psychiatric comorbidities was not extracted for the present secondary analysis.

Detailed sociodemographic characteristics of the full sample (*N* = 80) have been reported previously [33]. The analytic sample used for EGA comprised 73 participants with complete data on the selected EDE-Q items.

### Measures

#### Borderline Symptom List (BSL-95)

The BSL-95 [34] is a self-report instrument assessing the severity of BPD symptomatology. It consists of 95 items rated on a 5-point Likert scale ranging from 0 (“not at all”) to 4 (“very strong”) and was included to characterize symptom severity within the clinical sample.

#### Eating Disorder Examination Questionnaire (EDE-Q)

ED symptoms were assessed using the EDE-Q [19, 35], a self-report instrument derived from the Eating Disorder Examination (EDE; [36]) interview. It evaluates the severity and characteristics of ED psychopathology over the past 28 days and consists of 28 items. Twenty-two attitudinal items, assessing cognitive and emotional aspects of ED psychopathology, are rated on a 7-point scale ranging from 0 (“not on any day”) to 6 (“every day”), while six behavioural items assess the frequency of binge eating and purging behaviours. The EDE-Q comprises four subscales: *Restraint*, *Eating Concern*, *Weight Concern*, and *Shape Concern*. The EDE-Q is intended to assess eating disorder psychopathology and symptom severity and is not considered a diagnostic instrument [37].

### Statistical analyses

#### Descriptive analyses

Descriptive statistics were computed for all study variables, including means (*M*), standard deviations (*SD*), and ranges for continuous variables, as well as frequencies and percentages (%) for categorical variables. Internal consistency of multi-item scales was assessed using Cronbach’s alpha (*α*). All descriptive analyses were conducted using the analytic sample. The datasets used and/or analysed during the current study are available from the corresponding author on reasonable request.

At the item level, missingness was low (≤ 2 missing values per item), and no item exceeded a missingness threshold of 20%. No duplicate cases or extreme inter-item correlations (≥ 0.95) were identified. All items demonstrated sufficient variance and plausible value ranges, supporting their suitability for EGA.

#### Exploratory graph analysis

The dimensional structure of the attitudinal EDE-Q items was examined using EGA, a network-based approach that identifies dimensions as communities of densely connected items within a Gaussian graphical model (GGM). Analyses were restricted to theoretically relevant attitudinal EDE-Q items, including items assessing cognitive and emotional aspects of ED psychopathology (i.e., restraint, eating concern, weight concern, and shape concern). Items measuring behavioural frequencies (i.e., binge eating and purging behaviours) were excluded a priori because they use a different response format (frequency counts rather than Likert-type ratings) and are conceptually distinct from the attitudinal items. Accordingly, these items are typically reported descriptively rather than included in dimensional analyses of attitudinal ED psychopathology. Only complete cases were included in the primary analysis, and no imputation was performed to avoid introducing artificial associations. All items were converted to numeric format and standardized (*z*-scores) prior to network estimation.

All analyses were conducted in Python (version 3.12.4) using Jupyter Notebook, employing the packages pandas (version 3.0.1) [38] for data handling, numpy (version 1.26.4) [39] for numerical operations, scikit-learn (version 1.5.1) [40] for model estimation and regularization, igraph (Version 1.0.0) [41] and networkx (version 3.3) [42] for network construction and community detection, scipy (version 1.11.4) [43] for optimization routines, and matplotlib (version 3.9.0) [44] for visualization. To evaluate the robustness of the EGA results, a series of predefined sensitivity analyses was conducted following the analytic framework described by Laskowski et al., 2023 [27].

#### Network and dimensionality estimation

A GGM was estimated using graphical LASSO, which yields a sparse inverse covariance (precision) matrix representing regularized partial correlations between items. The regularization parameter (*α*) was selected via cross-validation (GraphicalLassoCV) and fixed across all bootstrap iterations to ensure comparability. The final model was estimated using this parameter with increased iteration limits to ensure numerical convergence. Partial correlations derived from the precision matrix were used as edge weights. Absolute values were used for network construction, while the sign of associations was retained for visualization. Dimensions were identified using the Walktrap [28] community detection algorithm, a random-walk–based method for detecting clusters of strongly interconnected nodes. The number of detected communities was interpreted as the number of dimensions.

#### Bootstrap stability and iterative item reduction

To assess the robustness of the identified structure, a nonparametric bootstrap procedure with 1,000 resamples was conducted. For each sample, the network was re-estimated and community detection repeated. Community labels were aligned using the Hungarian algorithm. Dimensional stability was quantified as the median number of dimensions across bootstrap samples with corresponding 95% percentile intervals.

Item stability was defined as the proportion of bootstrap samples in which an item was assigned to the same community as in the reference solution. Items with stability values below 0.70 were iteratively removed. After each step, EGA and bootstrapping were repeated until all remaining items met the predefined stability criterion. The final solution thus represents a stable subset of items contributing to the dimensional structure.

#### Sensitivity analyses

To assess robustness to missing data, EGA was re-estimated after median imputation using the SimpleImputer class. Solutions were compared to the complete-case analysis using the adjusted Rand index (ARI). The GGM was re-estimated using lower (75%), original, and higher (125%) values of the regularization parameter. Agreement with the reference solution was assessed using ARI. Results from Walktrap were compared with those obtained using the Louvain algorithm. Agreement between solutions was quantified using ARI. A leave-one-item-out (LOO) procedure was conducted by iteratively removing each item and re-estimating the model. Results were compared to the full solution using ARI to identify potential single-item dependencies.

#### Network visualization

The final network was visualized for descriptive purposes. Nodes represent EDE-Q items and edges represent regularized partial correlations. Edge strength was reflected by width and transparency, and node colours correspond to identified dimensions. The visualization served descriptive purposes only.

## Results

### Sample characteristics and data quality

The analytic sample (*N* = 73) did not differ significantly from the full sample in age (*t* = 0.11, *p* = .911), BMI (*t* = 0.06, *p* = .952), gender distribution (*χ²* = 5.22, *p* = .073), or educational attainment (χ² = 0.17, *p* = .682).

The analytic sample consisted predominantly of women (84.9%, *n* = 62), with 13.7% men (*n* = 10) and one individual identifying as gender diverse (1.4%). The mean age was 27.57 years (SD = 9.6, range = 18–61), and the mean BMI was 26.96 kg/m² (SD = 7.3, range = 17.3–55.8). Regarding educational attainment, 43.8% of participants (*n* = 32) reported more than 12 years of education, whereas 28.8% (*n* = 21) reported fewer than 12 years; data were missing for 27.4% of cases. Prior outpatient psychotherapy before DBT treatment was reported by 43.8% of participants (*n* = 32), while 39.7% (*n* = 29) indicated no prior psychotherapy; data were missing for 16.4% of cases.

### Questionnaire descriptive data

Distributions were inspected to ensure sufficient variance and valid response ranges; no items showed zero variance or invalid values. In the analytic sample, the EDE-Q attitudinal items included in the EGA demonstrated excellent internal consistency (*α* = 0.97). The mean score across these items was 3.13 (*SD* = 1.8, range = 0.0–5.8).

For descriptive sample characterization, the BSL-95 also showed excellent internal consistency (*α* = 0.97), with a mean score of 2.1 (*SD* = 0.6, range = 0.5–3.4).

### Exploratory graph analysis

#### Baseline dimensional structure

The baseline EGA was conducted on the 22 attitudinal EDE-Q items. The regularization parameter selected via cross-validation was *α* = 0.21. Walktrap community detection yielded a four-dimensional structure. The four dimensions comprised the following item clusters:

1. Dimension included items 1–5.
2. Dimension included items 6, 10, 12, 20, 22, 24.
3. Dimension included items 7–9, 19, 21.
4. Dimension included items 11 and 23–28.

Bootstrap analysis with 1,000 resamples yielded a median dimensionality of four dimensions (95% CI: 2–6), indicating substantial variability in the dimensional solution. Consistent with this, item stability varied considerably across items.

### Item stability and iterative item reduction

Item stability estimates revealed that nine items did not meet the predefined stability threshold of 0.70 (items 24, 19, 23, 9, 22, 12, 6, 10, and 20) and were therefore removed. A second EGA was conducted on the remaining 13 items. The cross-validated regularization parameter was slightly lower (*α* = 0.19). This analysis identified a three-dimensional structure, with bootstrap results indicating a median of three dimensions (95% CI: 2–3). All remaining items exceeded the stability threshold, indicating a stable and well-defined dimensional solution. The final item set comprised 13 items (items 1–5, 7, 8, 11, 21, and 25–28).

#### Final dimensional structure

**Fig. 1:**
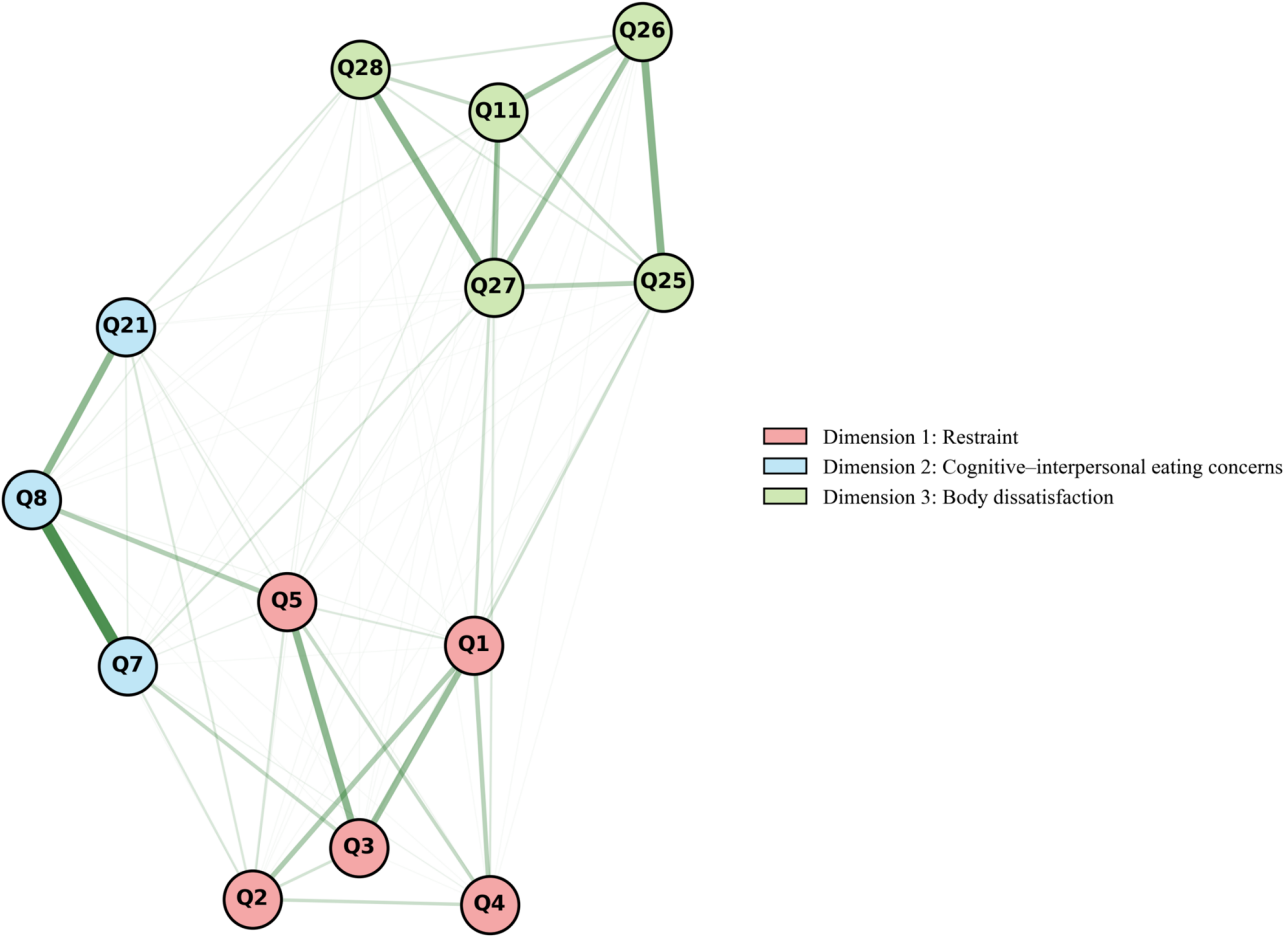
Final network structure of attitudinal EDE-Q items in patient with BPD derived from EGA. Note: Nodes represent individual questionnaire items, and edges represent regularized partial correlations estimated using a GGM with LASSO regularization (α = 0.19). Edge thickness and transparency reflect the magnitude of associations. Nodes are coloured according to the three identified dimensions: Dimension 1: Restraint (items 1–5), Dimension 2: Cognitive–Interpersonal Eating Concerns (items 7, 8, and 21), and Dimension 3: Body Dissatisfaction (items 11 and 25–28)

The final EGA solution identified three interpretable dimensions (see Fig. 1): *Restraint*, *Cognitive–Interpersonal Eating Concerns*, and *Body Dissatisfaction*. The first dimension, *Restraint* (items 1–5), reflects restrictive eating and dietary control, capturing deliberate efforts to limit food intake, adherence to dietary rules, and avoidance of eating. The second dimension, *Cognitive–Interpersonal Eating Concerns* (items 7, 8, and 21), is characterized by persistent thoughts about eating and body-related aspects, as well as concerns about eating in the presence of others. The third dimension, *Body Dissatisfaction* (items 11 and 25–28), reflects negative body-related affect and the importance of body-related criteria for self-worth.

### Sensitivity analyses

Re-estimation of the model using median imputation yielded an identical three-dimensional structure and identical item assignments (ARI = 1.00). Similarly, varying the regularization parameter (*α* = 0.16, 0.21, 0.26) did not affect the dimensional structure or item assignments (all ARI = 1.00). Comparison of community detection algorithms (Walktrap vs. Louvain) also resulted in identical solutions (ARI = 1.00), indicating that the findings were not dependent on the choice of clustering method. A LOO analysis demonstrated that the three-dimensional structure remained unchanged in 12 of 13 cases (ARI = 1.00). Only the removal of item 8 resulted in a reduced two-dimensional solution (ARI = 0.69), suggesting that this item may function as a connector within the network without substantially affecting the overall structure.

### Comparison with the original EDE-Q structure

As expected, the final EGA solution differed from the original four-factor structure of the EDE-Q. While the original *Restraint* factor was fully retained (items 1–5), the *Eating Concern* factor was reduced to a smaller subset of items reflecting cognitive preoccupation and social-evaluative aspects (items 7, 8, and 21). Items reflecting guilt, secrecy, and loss of control did not show sufficient stability in this sample. Furthermore, items from the original *Shape* and *Weight Concern* subscales converged into a single dimension reflecting body dissatisfaction and self-evaluation based on shape and weight (items 11 and 25–28). This dimension primarily captured affective and identity-related aspects, whereas items related to avoidance behaviours and appearance ideals were not retained.

## Discussion

Although the EDE-Q [19] is an established and widely used self-report measure of ED psychopathology, evidence regarding its construct validity and factor structure in specific clinical populations remains limited. The present study identified a reduced three-dimensional structure, comprising *Restraint*,* Cognitive-Interpersonal Eating Concerns*,* and Body Dissatisfaction*, suggesting that the organization of attitudinal ED psychopathology may differ from the original factor structure in the present population with BPD.

This is in line with previous research demonstrating limitations of the EDE-Q, particularly regarding the instability and limited reproducibility of its original four-factor structure, the limited discriminant validity of subscales, and an inconsistent measurement invariance across both non-clinical and clinical populations, with converging evidence from factor-analytic and network-based approaches [20, 21, 23, 26, 27, 45, 46]. In clinical samples of women with EDs, EGA has demonstrated that the original factor model is not stable across diagnostic groups and that alternative structures, typically comprising fewer and more homogeneous dimensions, provide a better representation of the data [27]. Jenkins et al. [26] further concluded that the original factor structure is rarely replicated and that the exclusion of poorly performing items is frequently supported across diverse populations.

Notably, the original *Eating Concern* subscale was reduced to a smaller subset in the present study, primarily reflecting cognitive preoccupation and social-evaluative concerns. This is consistent with previous findings indicating that *Eating Concern* items are among the least stable components of the EDE-Q and are often not retained as a distinct dimension in abbreviated or alternative models [21, 46, 47]. Similarly, our results converge with prior studies reporting a merging of *Shape and Weight Concern* items into a combined dimension [23, 48–50], corresponding to the *Body Dissatisfaction* dimension identified in the present EGA. Factor-analytic work in male clinical samples has likewise demonstrated substantial deviations from the original EDE-Q structure, including altered factor compositions and exclusion of multiple items due to low communalities [20]. These findings suggest that the factorial validity of the EDE-Q is context-dependent and sensitive to sample characteristics and underlying psychopathology.

At the same time, several characteristics associated with BPD may be relevant when interpreting the altered organization of body-related concerns observed in the present study. Previous research suggests that body image disturbances in BPD occur independently of objective weight status and are only partially explained by co-occurring EDs [51–53]. Individuals with BPD often report markedly negative attitudes toward their bodies, low satisfaction with their physical appearance, and heightened discomfort related to body image [51, 53]. In addition, physical attractiveness and external evaluation may hold particular relevance for self-esteem and perceived self-worth in this population [54]. These findings suggest that body-related concerns in BPD may extend beyond classical weight- or shape-focused ED psychopathology and instead may partly reflect broader self-evaluative and interpersonal processes [55, 56].

In line with this interpretation, *Body Dissatisfaction* emerged as a particularly central and coherent dimension in the present study. This dimension comprised items related to feelings of fatness, dissatisfaction with body shape or weight, distress related to body perception, and the importance of appearance-related criteria for self-evaluation. Similar *Body Dissatisfaction* dimensions have been identified across EDE-Q studies, including previous factor-analytic and EGA analyses showing overlap between *Shape* and *Weight Concern* items [48–50]. Furthermore, recent findings from large transdiagnostic clinical samples suggest that ED psychopathology may be best represented along a single broad dimension, with body dissatisfaction and weight/shape concerns emerging as especially central features [57].

However, while *Body Dissatisfaction* is consistently observed across ED research, its content may differ depending on the population under investigation. In classical ED samples, body dissatisfaction typically encompasses behavioural and cognitive aspects such as body checking, avoidance, and overevaluation of shape and weight. In contrast, the present findings suggest that, in individuals with BPD, the distinction between shape and weight concerns may be less clearly differentiated. Previous research indicates that body image disturbances are common in BPD and are only partially explained by co-occurring EDs [17, 51]. Individuals with BPD frequently report negative body-related self-evaluations, low body satisfaction, and heightened body image disturbance [17, 51, 53, 54]. Accordingly, body dissatisfaction in this population may reflect broader difficulties in self-concept and self-evaluation rather than isolated concerns about body shape or weight [17, 54].

This interpretation is further supported by findings indicating that body image disturbance and negative body-related self-evaluations in BPD may be linked to broader difficulties in self-concept and self-esteem [17, 54]. Reduced differentiation at the level of self-concept may contribute to overlapping or less distinct factor structures within the EDE-Q and may help explain why *Shape* and *Weight Concern* items converged into a single Body Dissatisfaction dimension in the present study. However, this interpretation remains tentative, as the present study did not directly assess self-concept, self-esteem, or related constructs that could clarify the mechanisms underlying the observed factor structure.

A further potential contributing mechanism may involve altered interoceptive processing in BPD. Prior research suggests that individuals with BPD may show reduced awareness of internal bodily sensations during conscious attention while relying more strongly on external cues for emotional and behavioural regulation [58]. Reduced interoceptive integration may represent one potential mechanism contributing to less differentiated representations of body-related cognitions and could partly explain the overlapping body-related dimensions observed in the present EDE-Q structure. This interpretation is consistent with theoretical models and empirical findings emphasizing identity disturbance, unstable self-image, and emotional reactivity as core features of BPD [1, 17, 56].

The *Restraint* dimension was fully replicated and highly stable, mirroring one of the most robust findings across ED research. Restrictive eating and dietary control therefore appear to represent a transdiagnostic and structurally coherent component of ED psychopathology. In contrast, *Eating Concern* did not emerge as an independent dimension but was reduced to a narrower cognitive–interpersonal core. Specifically, items reflecting preoccupation with eating and concerns about eating in the presence of others were retained, whereas items capturing guilt, secrecy, and loss of control were unstable and excluded. One possible explanation is that broader disturbances in self-concept may attenuate the relative contribution of eating-related concerns to overall self-evaluation. Individuals with a globally negative self-view may report low relative importance of weight or shape, not because such concerns are absent, but because their self-esteem is already broadly impaired across multiple domains. In such cases, eating- or body-related cognitions may become less distinguishable from pervasive negative self-evaluations. This fragmentation of *Eating Concern* is consistent with previous findings suggesting that this dimension is particularly sensitive to sample characteristics and may not represent a stable or unified construct [47].

The present findings suggest that attitudinal ED psychopathology in individuals with BPD may not fully conform to the original EDE-Q factor structure and may be organized in a manner that places greater emphasis on body-related concerns and self-evaluative aspects [11, 18]. Within this framework, *Body Dissatisfaction* emerged as a central organizing dimension that may extend beyond isolated concerns about body shape or weight.

The findings have important methodological and clinical implications. First, the routine use of standard EDE-Q subscale scores in BPD samples may be problematic, as these scores may not reflect valid underlying constructs. Second, the consistent emergence of a *Body Dissatisfaction* dimension across studies highlights its potential as a transdiagnostic target for future research and intervention. Third, affective and self-evaluative aspects of body dissatisfaction may warrant further investigation in relation to ED psychopathology in individuals with BPD. These dimensions could reflect broader cognitive-affective features of disordered eating alongside other classical ED symptoms such as body image disturbance and negative self-evaluation. Finally, the findings underscore the importance of validating assessment instruments within specific clinical populations and support the use of network-based approaches such as EGA, which allow for a more flexible and empirically grounded identification of dimensional structures.

Several limitations should be considered. First, the sample size was relatively small, which limit the generalizability of the findings. However, the stability of the final solution within the present dataset was supported by robustness analyses, including bootstrap resampling, parameter variation, and LOO procedures, all of which yielded highly consistent results. Second, the sample consisted of individuals with BPD undergoing DBT, predominantly from inpatient settings and largely female, which restricts generalizability to other populations. Furthermore, participants completed the questionnaires at varying stages of treatment. As symptom severity and symptom interrelations may change during the course of DBT, the observed dimensional structure may partly reflect variation in treatment stage.

Third, although participants underwent routine clinical assessment as part of their treatment, information on formal eating disorder diagnoses was not extracted for the present secondary analysis. Consequently, the present findings are based exclusively on self-reported eating disorder psychopathology assessed with the EDE-Q, and conclusions regarding specific eating disorder diagnoses cannot be drawn. In addition, information on psychiatric comorbidities beyond BPD (e.g., PTSD, anxiety disorders, or depressive disorders) was not available for the present secondary analysis. Therefore, it remains unclear to what extent the identified EDE-Q structure may have been influenced by co-occurring psychopathology rather than BPD alone. Furthermore, the present study did not examine associations between the identified EDE-Q dimensions and external measures of BPD symptom severity or related constructs, limiting conclusions regarding convergent validity and the broader clinical interpretation of the identified structure. Fourth, the cross-sectional design precludes conclusions regarding temporal stability or predictive validity of the identified dimensions. Finally, only attitudinal EDE-Q items were included, limiting conclusions regarding behavioural aspects of ED pathology. Despite these limitations, the convergence of findings across multiple analytic approaches and sensitivity analyses strengthens confidence in the robustness of the identified dimensional structure.

## Conclusions

The findings suggest that the EDE-Q does not retain its original four-factor structure in individuals with BPD. In the present sample, a three-dimensional solution emerged, comprising Restraint, Cognitive-Interpersonal Eating Concerns, and Body Dissatisfaction. Several aspects of this structure are consistent with previous factor-analytic and network-based studies reporting deviations from the original EDE-Q model, including a merging of Shape and Weight Concern items across diverse populations. However, the present findings also suggest that the organization of attitudinal ED psychopathology in individuals with BPD may not fully conform to the original EDE-Q structure. Given the exploratory nature of the study and the absence of external validators, interpretations regarding the mechanisms underlying the observed structure remain tentative. The findings therefore support a cautious interpretation of standard EDE-Q subscale scores in BPD populations and underscore the importance of validating assessment instruments within specific clinical populations.

## Data Availability

The datasets used and/or analysed during the current study are available from the corresponding author on reasonable request.

## Abbreviations

AN: Anorexia nervosa
ARI: Adjusted Rand index
BED: Binge-eating disorder
BMI: Body mass index
BN: Bulimia nervosa
BPD: Borderline personality disorder
BSL-95: Borderline Symptom List
CI: Confidence interval
DBT: Dialectical Behaviour Therapy
DSM-V: Diagnostic and Statistical Manual of Mental Disorders
ED: Eating disorders
EDE: Eating Disorder Examination
EDE-Q: Eating Disorder Examination Questionnaire
EDNOS: ED not otherwise specified
EGA: Exploratory Graph Analysis
GGM: Gaussian graphical model
LOO: Leave-one-item-out
M: Means
SD: Standard deviations
